# Growth and Photovoltaic Properties of High-Quality GaAs Nanowires Prepared by the Two-Source CVD Method

**DOI:** 10.1186/s11671-016-1420-y

**Published:** 2016-04-12

**Authors:** Ying Wang, Zaixing Yang, Xiaofeng Wu, Ning Han, Hanyu Liu, Shuobo Wang, Jun Li, WaiMan Tse, SenPo Yip, Yunfa Chen, Johnny C. Ho

**Affiliations:** State Key Laboratory of Multiphase Complex Systems, Institute of Process Engineering, Chinese Academy of Sciences, Beijing, 100190 China; Center for Excellence in Urban Atmospheric Environment, Institute of Urban Environment, Chinese Academy of Sciences, Xiamen, 361021 China; Department of Physics and Materials Science, City University of Hong Kong, Kowloon, Hong Kong; State Key Laboratory of Millimeter Waves, City University of Hong Kong, Kowloon, Hong Kong; Shenzhen Research Institute, City University of Hong Kong, Shenzhen, 518057 China; Beijing National Day School, Beijing, 100039 China

**Keywords:** GaAs, Chemical vapor deposition, Two-source, Contact printing, Nanowire parallel arrays, Schottky solar cells, 73.21.Hb, 78.56.-a, 81.05.Ea

## Abstract

Growing high-quality and low-cost GaAs nanowires (NWs) as well as fabricating high-performance NW solar cells by facile means is an important development towards the cost-effective next-generation photovoltaics. In this work, highly crystalline, dense, and long GaAs NWs are successfully synthesized using a two-source method on non-crystalline SiO_2_ substrates by a simple solid-source chemical vapor deposition method. The high V/III ratio and precursor concentration enabled by this two-source configuration can significantly benefit the NW growth and suppress the crystal defect formation as compared with the conventional one-source system. Since less NW crystal defects would contribute fewer electrons being trapped by the surface oxides, the p-type conductivity is then greatly enhanced as revealed by the electrical characterization of fabricated NW devices. Furthermore, the individual single NW and high-density NW parallel arrays achieved by contact printing can be effectively fabricated into Schottky barrier solar cells simply by employing asymmetric Ni-Al contacts, along with an open circuit voltage of ~0.3 V. All these results indicate the technological promise of these high-quality two-source grown GaAs NWs, especially for the realization of facile Schottky solar cells utilizing the asymmetric Ni-Al contact.

**Electronic supplementary material:**

The online version of this article (doi:10.1186/s11671-016-1420-y) contains supplementary material, which is available to authorized users.

## Background

Due to the direct, suitable bandgap (1.42 eV) and its superior high carrier mobility (8000 cm^2^/Vs for electrons, 400 cm^2^/Vs for holes), GaAs materials possess a maximum theoretical single-junction photon-to-electricity conversion efficiency of ~30 %; therefore, their nanowire (NW) materials are widely adopted as fundamental building blocks for next-generation electronics and photovoltaics [1–5]. For example, Colombo et al. demonstrated a single GaAs NW radial p-i-n solar cell with an efficiency of 4.5 % [6], and Holm et al. later realized a similar single GaAs NW solar cell with an efficiency of 10.2 % after surface passivation by GaAsP shells [7] under air mass (AM) 1.5G illumination. Until recently, Krogstrup and his group fabricated the vertically aligned single GaAs NW solar cell with an efficiency over the Shockley-Queisser limit of ~40 % due to the light concentration effect of the NWs with diameters comparable with the incident photon wavelength [8]. Although the illustrated efficiency is promising, the associated fabrication and material cost require a significant reduction in order to meet the requirements of third-generation cost-effective photovoltaics.

Generally, most of the GaAs NWs reported in the literature are grown by molecular beam epitaxy (MBE) or metalorganic chemical vapor deposition (MOCVD) methods via the vapor-liquid-solid (VLS) and/or vapor-solid-solid (VSS) mechanism [1, 9, 10]. The equipment employed in these technologies is typically complicated, and the required single-crystalline growth substrates are expensive, accounting for the substantial cost of NW synthesis. Meanwhile, most of the reported solar cells are always configured into p-i-n junctions, which necessitate the finely controlled growth conditions and device fabrication for reliable high-quality junction formation and metallization [6–8, 11, 12]; therefore, it is highly desired to develop low-cost alternative methods to grow crystalline GaAs NWs in the large scale, as well as to develop a facile process to fabricate solar cell devices in the simple structure. For example, Dhaka et al. prepared high-quality GaAs NWs on glass substrates, but neither solar cell devices nor the corresponding electrical property was fabricated and characterized, respectively [13]. Even though Yang et al. reported employing a cost-effective Schottky contact for the large-scale preparation of carbon nanotube (CNT) solar cells, the obtained efficiency is not acceptable with only 0.11 % at an illumination of 90 kW/cm^2^ due to the small bandgap of CNTs [14].

In the previous reports, we have achieved long and high-quality GaAs NWs on a non-crystalline SiO_2_ substrate by a two-step CVD method where a high-temperature nucleation step is needed to obtain the higher supersaturated Ga concentration in Au seeds before the NW growth at a relatively lower temperature. The growth procedure is somewhat complicated, and special care should be taken to control temperature profiles especially in the heating and cooling steps [15, 16]. We also fabricated Schottky-contacted GaAs NW solar cells with Au catalyst tips [17]. Although the efficiency is demonstrated with ~2.8 % for the single NW device, large-scale integration of these GaAs NWs with well-aligned Au-Ga catalysts is difficult and has not succeeded till now. In this study, we prepare GaAs NWs by a facile two-source method in the one-step growth platform. The NWs can be grown longer with high crystallinity as compared with those grown by the one-source method. The long NWs can then facilitate the contact printing for the fabrication of high-density NW arrays and the subsequent fabrication of Schottky barrier solar cells with Ni-Al asymmetric contacts, which are promising for the next-generation, high-efficiency, low-cost solar cells.

## Methods

### GaAs NW Synthesis and Characterization

**Fig. 1:**
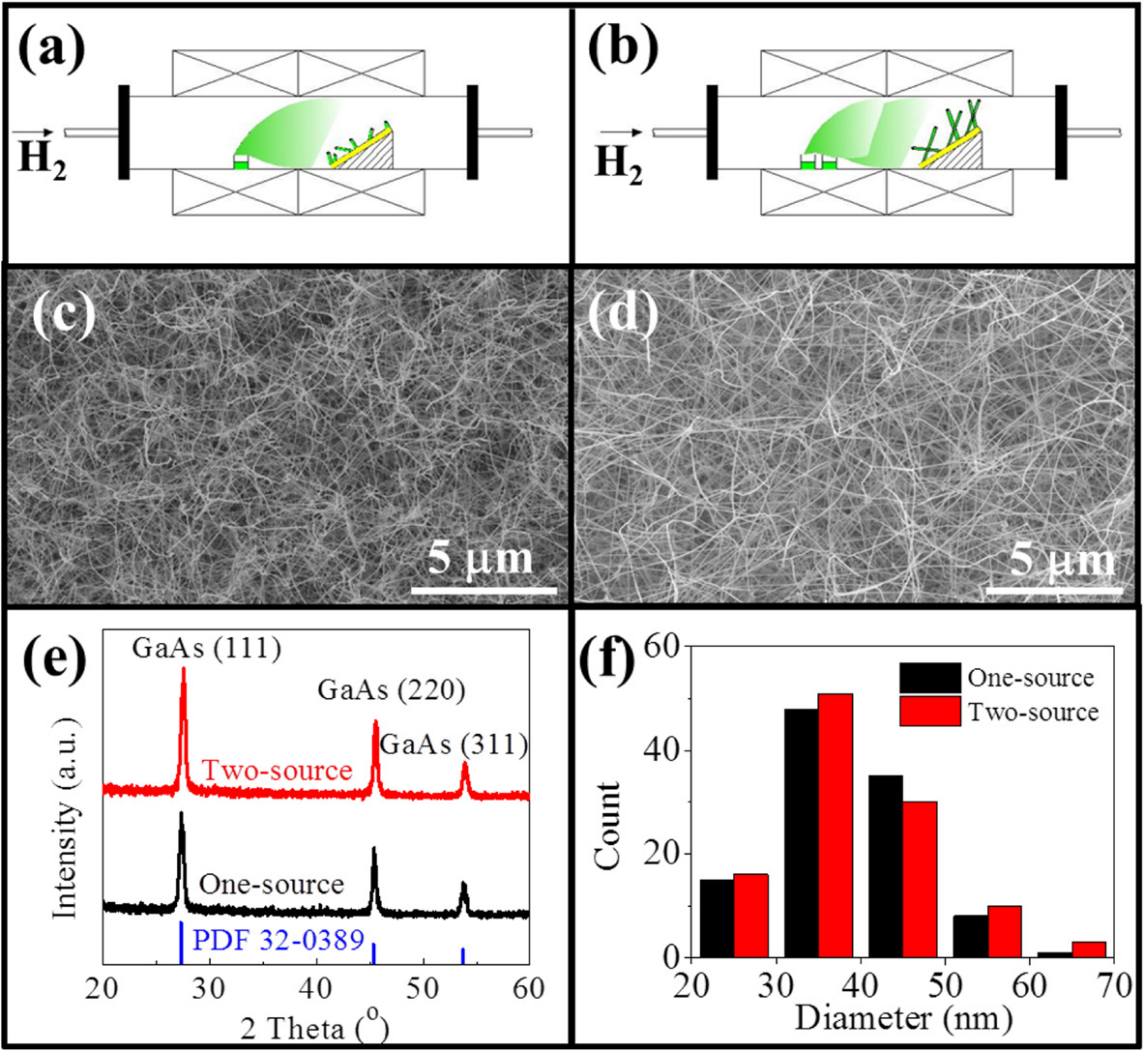
Comparison between the one-source and two-source growth methods. **a**, **c** Growth schematics and SEM of GaAs NWs grown by the one-source configuration. **b**, **d** Growth schematics and SEM of GaAs NWs grown by the two-source configuration. **e** XRD patterns. **f** NW diameter distribution histograms

The GaAs NWs are synthesized in a solid-source CVD system using the annealed Au thin film as catalytic seeds as reported previously [18, 19]. In detail, the boron nitride crucible holding GaAs powders (~1 g, 99.9999 % purity) and the SiO_2_/Si substrates (50-nm-thick thermal oxide) with the thermally deposited Au film (2.5 nm in nominal thickness) are placed in the centers of the upstream and downstream zones of a two-zone tube furnace, respectively, serving as the solid source and the growth catalyst. After the system is pumped down to ~10^−3^ Torr, H_2_ gas (100 standard cubic centimeters per minute, sccm, 99.999 % purity) is provided through the control of the mass flow controller, and the Au catalyst film is then annealed into nanoparticles at 800 °C for 10 min. After the catalyst zone is cooled down to the growth temperature (600 °C), the upstream zone starts to ramp up to 850 °C to evaporate the GaAs powders, and the vapor precursors are then transported by H_2_ to the downstream zone for the NW growth. After a growth duration of 60 min, the system is cooled down to room temperature in H_2_ atmosphere, and then the NWs are harvested for subsequent characterization and device fabrication. The only difference between this two-source growth performed here and the conventional method is that two crucibles (the second crucible is approximately 2 cm away from the first one) holding extra GaAs powder sources are used with a total weight of ~2 g in the source zone with the setup schematics shown in Fig. 1a and b.

Surface morphologies of the grown GaAs NWs are examined by scanning electron microscopy (SEM, FEI/Philips XL30). Crystal structures are determined by collecting X-ray diffraction (XRD) patterns on a Philips powder diffractometer using Cu Kα radiation (*λ* = 1.5406 Å) and by high-resolution transmission electron microscopy (HRTEM, JEOL 2100F). For the HRTEM analysis, the GaAs NWs are first suspended in absolute ethanol by ultrasonication and then drop-casted onto a copper grid for the corresponding characterization.

### GaAs NW Device Fabrication and Measurement

The GaAs NW arrays are fabricated by contact printing on SiO_2_/Si substrates (50-nm-thick thermally grown oxide) as reported previously [20, 21]. Typically, a pre-patterned SiO_2_/Si substrate is coated with photoresist to serve as the receiver, while the donor NW chip is flipped onto the receiver and slid at a rate of 20 mm/min with a pressure of 20 g/cm^2^. After the photoresist is removed, the GaAs NW arrays are then left on the patterned region, where conventional photolithography is utilized to define the electrodes and the 70-nm-thick Ni film is thermally deposited as the contact electrodes followed by a lift-off process in order to realize the NW field-effect transistors (FETs).

The single GaAs NW solar cell is also fabricated by photolithography. Firstly, NWs are dispersed in absolute ethanol by ultrasonication, and the dispersion is then drop-casted onto the SiO_2_/Si substrates. Next, two successive photolithographies are carried out to define the first Ni layer and the second Al layer by the careful alignment with the controllable channel length. Similarly, the GaAs NW array solar cells are fabricated by contact printing and the two consecutive photolithographic steps in order to define the Ni-Al contact.

Electrical performances of the NW array FETs and solar cells are characterized with a standard electrical probe station and an Agilent 4155C semiconductor analyzer. For the solar cell testing, the illumination is provided by using a solar simulator (Newport 96000) with an intensity of AM 1.5G (100 mW/cm^2^).

## Results and Discussion

**Fig. 2:**
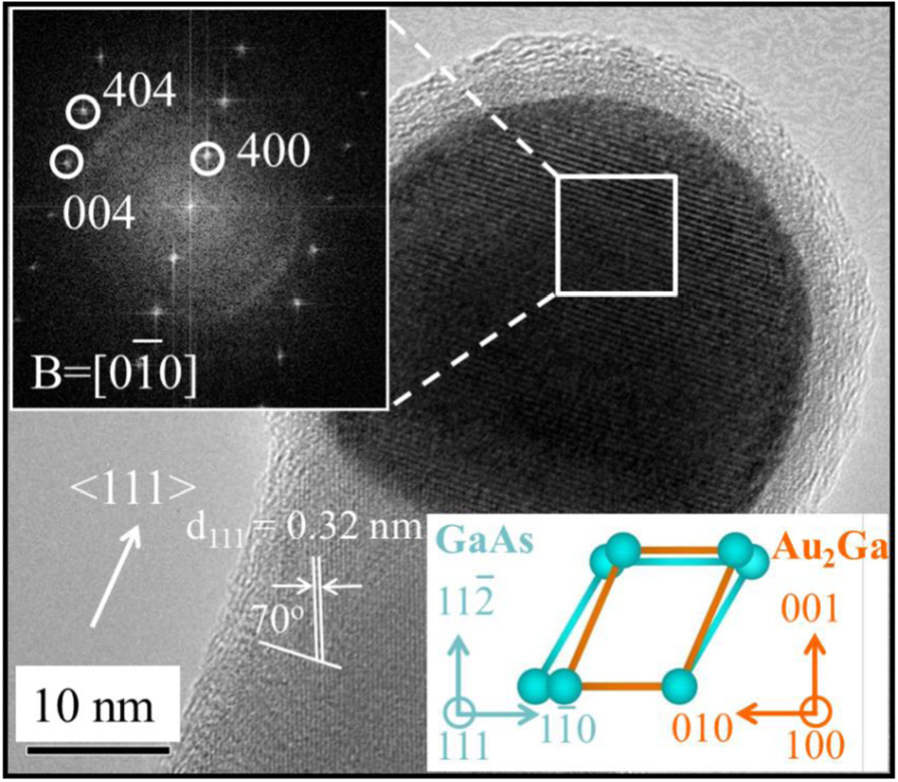
HRTEM image, the corresponding FFT of the Au-Ga catalytic alloy tip, and the Ga atom alignment in the Au_2_Ga/GaAs NW interface of one typical GaAs NW grown by the two-source technique

In order to compare and contrast our two-source method versus the conventional single-source CVD growth, the growth system schematics as well the resulting morphology of GaAs NWs are shown in detail in Fig. 1. It is clear from the SEM images that the GaAs NWs grown by the two-source method are straighter and longer as illustrated in Fig. 1c, d. As displayed in Fig. 1e, all the obtained NWs existed in the cubic zinc-blende phase identified by XRD patterns, in accordance to the standard card PDF 32-0398. The diameter distribution is then investigated by measuring the diameter of more than 100 NWs, where the histograms show that a similar diameter distribution is attained with a mean value of 39 nm employing both single- and two-source methods. Specifically, one representative NW grown in the two-source system is further analyzed by HRTEM in Fig. 2. The catalyst tip is clearly shown in the image, and after fast Fourier transformation (FFT) and energy-dispersive X-ray spectroscopy (EDS) analysis, the catalyst tip is composed of the Au_2_Ga crystal phase. Furthermore, the NW is grown along the <111> direction with two (111) planes intersecting with an angle of ~71°, having the epitaxy relationship of the Au_2_Ga (400) plane parallel to the GaAs (111) plane. This result is perfectly consistent with our previous report that NWs with a diameter of ~30 nm would have the catalytic tip of Au_2_Ga with the Ga supersaturation ~33.3 atm.% in the Au seeds due to the Gibbs-Thomson effect [19]. As depicted in the inset of Fig. 2, the Au_2_Ga(400)||GaAs(111) interface is found to have minimal lattice mismatch [(*a*_NW_ − *a*_CAT_)/*a*_NW_ × 100%, 20 % for GaAs<110>|Au_2_Ga<010>, and −10 % for GaAs<112>|Au_2_Ga<001>] among all possible Ga atomic configurations there, which would justify the preferred epitaxial growth of GaAs NWs from the Au_2_Ga catalyst seeds. It is also noticed that the Au catalyst tips have the merits of crystallinity as identified by electron diffraction, especially in the tip/NW interface region as revealed by surface X-ray diffraction [10, 22], making the Au catalyst quasi-solid although it existed in a spherical shape as observed by TEM. All these results reveal that the GaAs NWs, grown by the two-source method, are grown via the VSS mechanism, which is in good agreement with the literature [10, 23]. Importantly, high-quality, long, and dense GaAs NWs are as well successfully synthesized on the non-crystalline SiO_2_ substrate here.

To shed light on the advantage and characteristics of this two-source growth, we would first focus on the operation of our CVD system. During the NW growth, the GaAs source powder is decomposed and evaporated into Ga and As_2_ species to serve as the gas phase precursors. Due to the relatively higher vapor pressure of As_2_ than Ga, more As_2_ species get evaporated from the powder surface as compared with the ones of Ga. This way, a high V/III ratio resulted, in which this ratio would decrease eventually in the prolonged growth since the As_2_ species would get depleted faster in the source [24, 25]. In this context, the additional crucible carrying the source powder, located at a relatively lower temperature (i.e., ~20 °C) due to the temperature gradient in the furnace, will not only give rise to the initial higher V/III ratio but also help to sustain a constant magnitude of this ratio. Notably, this stable V/III ratio is found to play a key role in most of the III-V NW growth [26–29]. For example, the higher growth rates and better crystal quality of GaAs NWs have been readily obtained by providing the higher V/III ratio along with the enhanced precursor amount in the MOCVD system [27]. By solely tuning the V/III ratio, cubic zinc-blende and hexagonal wurtzite phases can be finely tailored for most of the III-V NW growth [28]. All these have evidently revealed that this two-source method is exceedingly beneficial for the facilitation of a high V/III ratio required for the high-quality GaAs NW growth environment.

**Fig. 3:**
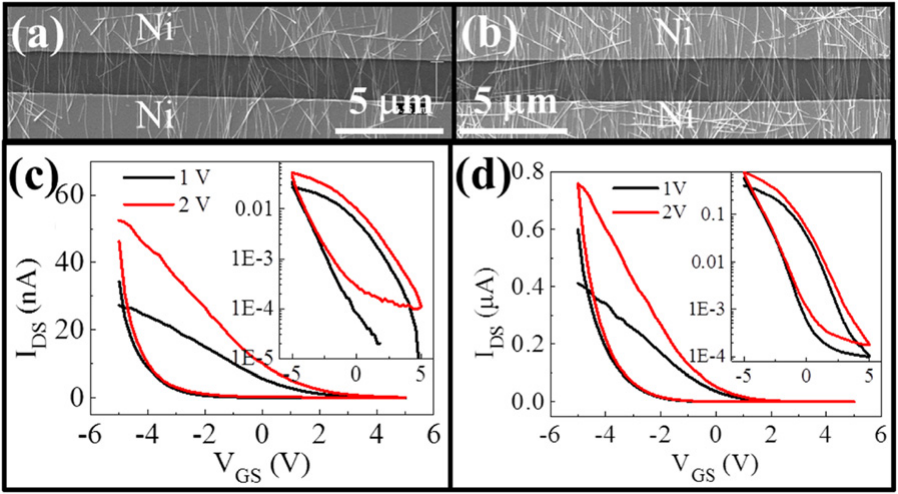
Comparison of the electrical properties of the GaAs NW parallel array FETs fabricated with NWs grown by different techniques. **a**, **c** SEM and *I*_DS_-*V*_GS_ curves of devices constructed with NWs grown by the one-source configuration. **b**, **d** SEM and *I*_DS_-*V*_GS_ curves of devices configured with NWs grown by the two-source technique

In order to further explore their electrical and photovoltaic properties, the obtained GaAs NWs are then integrated into parallel arrays by contact printing and next fabricated into FETs using Ni as the optimized electrodes for the ohmic contact of intrinsically p-type GaAs NW devices [18, 30]. Figure 3 gives the SEM images and transfer curves of the fabricated NW devices. As depicted in Fig. 3a and b, it is clear that by employing the same NW printing condition, the NW print density of the one-source grown samples is only 2–3/μm, while the density is enhanced to 4–6/μm for the two-source grown ones due to the longer grown NWs here. These longer NWs can enable the improved printing by aligning, attaching, and detaching the NW more effectively on the substrate for the higher printing density. Figure 3c and d displays the corresponding *I*_DS_-*V*_GS_ curves, where the p-type conductivity is yielded with a high ON/OFF ratio of up to 10^3^–10^4^ for all samples, and the ohmic contact is further confirmed by the *I*_DS_-*V*_DS_ curves as shown in Additional file 1: Figure S1. It should be noted that the representative high-density NW array FET (i.e., two-source grown sample) has a high equivalent ON current of 0.4 nA/NW, far higher than the low-density one (i.e., one-source grown sample) of merely 0.08 nA/NW considering the channel width of 200 μm. This higher ON current can be probably attributed to the enhanced NW crystal quality. In our previous studies, it is found that the p-type conductivity comes from the surface oxide layer, where the abundant trap states deplete the electrons in the GaAs NW core [30]. Therefore, the enhanced crystal quality here would give fewer free electrons, and thus, more holes are generated by the surface oxide layers. A similar phenomenon is also observed in the high-quality GaAs NWs by the two-step growth method as reported in the past [15]. As a result, these long NWs with the improved crystal quality not only benefit the contact printing for the high-density NW array integration but also are promising for photovoltaics, as less crystal defects would make the photon-induced minority carrier have a longer lifetime for the higher photocurrent.

**Fig. 4:**
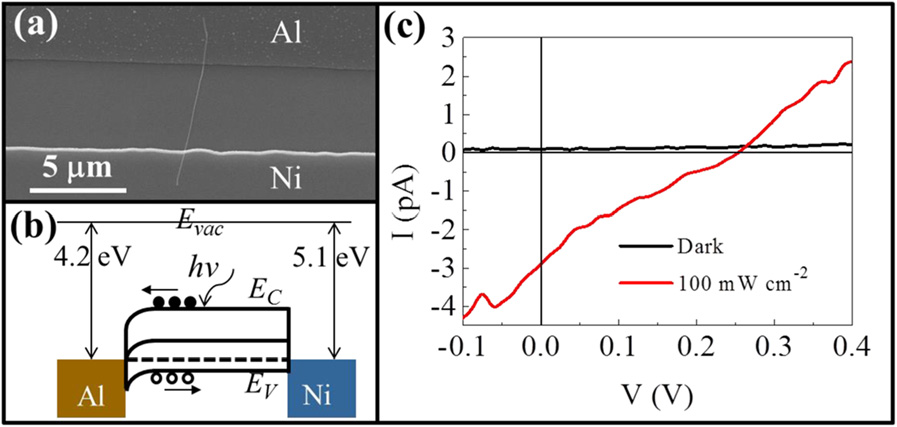
Photovoltaic characteristics of the GaAs NW Schottky devices. **a** SEM image, **b** band diagram alignment, and **c** the solar cell performance in the dark and under 1 sun illumination

Demonstrating the proof-of-concept as well as avoiding the complicated p-i-n NW junction formation for low-cost solar cells, Schottky NW solar devices are developed in this work by employing the asymmetric Ni-Al contact with a work function of 5.1 and 4.2 eV, respectively. Figure 4a and b illustrates the SEM image and band alignment of the single GaAs NW solar cell, while Fig. 4c shows the corresponding *I*-*V* characteristics. It is obvious that the asymmetric Ni-Al Schottky contact works well for the solar cell, with an open circuit voltage *V*_OC_ = 0.27 V and *I*_SC_ = 3 pA under an illumination of 100 mW/cm^2^. Considering the channel length of ~5.5 μm and the NW diameter of ~38 nm, the energy conversion efficiency is estimated to be ~0.1 %. This efficiency is not impressive due to the fact that the diameter is too small leading to the insufficient absorption of incident phonons. Also, the channel length might be larger than the minority diffusion length such that the carrier collection efficiency gets degraded. In specific, the minority diffusion length (*l*) can be estimated to be ~1 μm by the equation *l* = (*μτkT*/*e*)^1/2^, where *μ* is electron mobility of ~4000 cm^2^V^−1^s^−1^, *τ* is electron lifetime in the order of 10^2^ ps [31, 32], and *kT*/*e* is a constant. In the future, the contact resistance can also be further reduced in order to enhance the fill factor. It is also noted that the obtained *V*_OC_ is much lower than the work function difference of 0.9 eV for the asymmetric Ni-Al contact here, which is probably attributed to the Fermi level pinning effect of the III-V materials, making the Schottky barrier lower than the theoretically calculated one [33–35]. For more than 30 devices characterized, the mean *V*_OC_ is 0.32 ± 0.08 V, which is similar to our previous report [36].

**Fig. 5:**
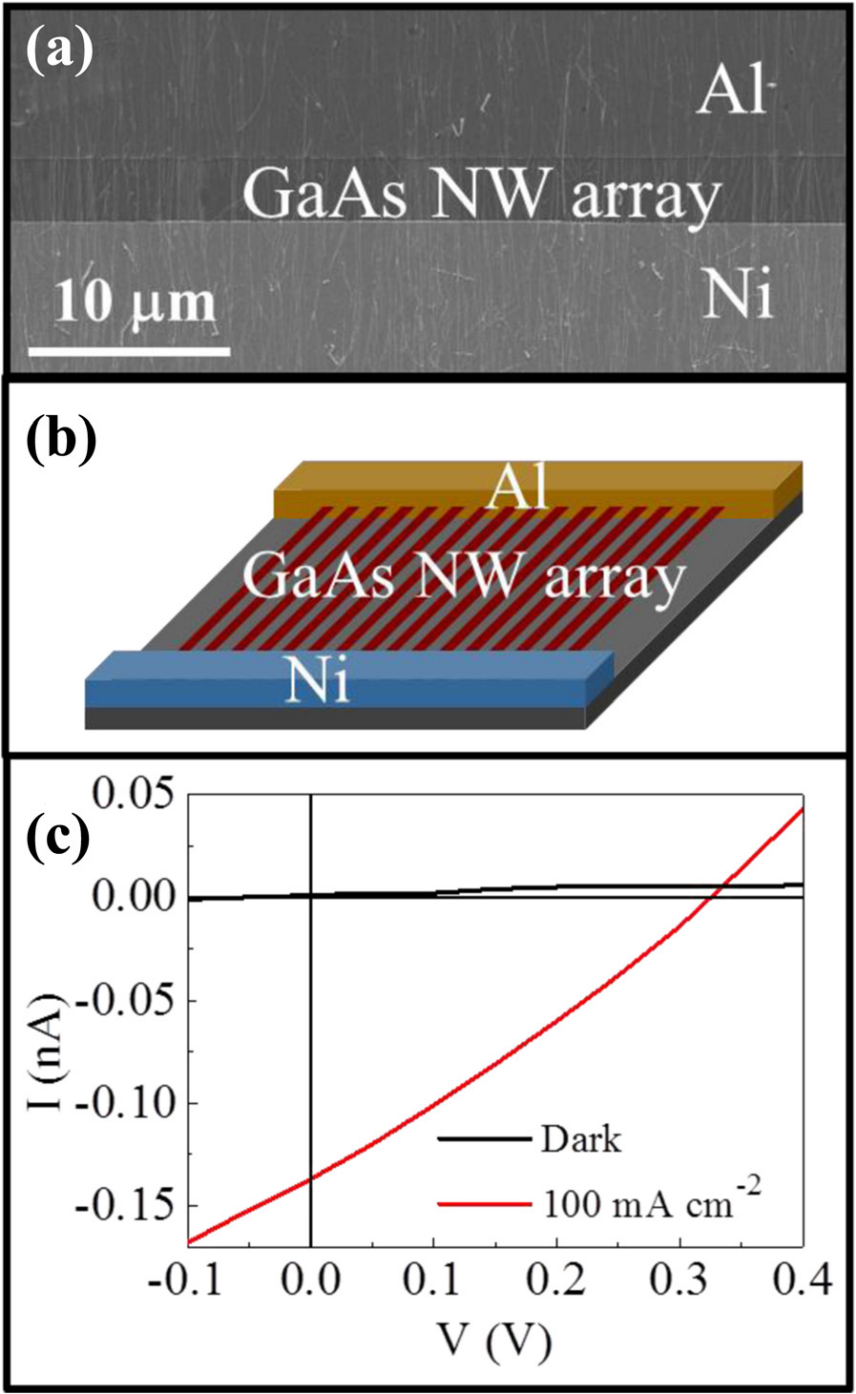
Photovoltaic behaviors of the GaAs NW array Schottky devices. **a** SEM image, **b** device schematic, and **c** *I*-*V* curves (photovoltaic performance) of the representative NW array solar cell fabricated by NW contact printing

In any case, to further evaluate the advantages of these long NWs grown by the two-source method, and to seek for possible applications in the large-scale solar cells, these long NWs are contact printed into high-density NW arrays and the asymmetric Ni-Al Schottky contact is as well defined to fabricate the NW array solar cells. As shown in the SEM image and corresponding *I*-*V* behavior of the high-density NW solar cell in Fig. 5a and b, respectively, the observed *V*_OC_ is slightly enhanced to ~0.32 V, while *I*_SC_ is as large as 140 pA, indicating the integrated effect of the NW parallel arrays. Although the obtained energy conversion efficiency is not respectively high due to the low photon absorption efficiency coming from the thin NW thickness of only 30–40 nm, this facile solar cell fabrication still holds the promise for the improved efficiency by further optimizing the Schottky barrier height, active channel length, NW diameters, and NW print density achieved through multi-layer contact printing [37].

## Conclusions

In conclusion, high-quality, long, and dense GaAs NWs are synthesized by a facile two-source chemical vapor deposition method. The grown NWs are found to have better crystallinity as well as retain the same cubic zinc-blende phase and diameter distribution, via the vapor-liquid-solid growth mechanism as compared with those grown by the conventional one-source technique. Specifically, fewer crystal defects and longer NW length resulted, probably attributable to the higher V/III ratio and precursor concentration enabled by this two-source configuration, which is in good agreement with the literature. When fabricated into NW parallel array FET devices, the enhanced NW crystal quality together with the higher NW print density can contribute improved performance to their p-type conductivity. At the same time, the asymmetric Ni-Al Schottky contact works well for both single NW and NW parallel photovoltaic devices. All these results indicate the successful synthesis of high-quality GaAs NWs by tailoring the V/III ratio in the two-source growth method, and importantly the potential applications in the area of facile NW Schottky solar cells, illustrating their promising potency for next-generation photovoltaics.

## Additional file

Additional file 1: Figure S1.
*I*_DS_-*V*_DS_ curves of the GaAs NW array FET illustrated in Fig. 3b of the main text. The curves confirm the ohmic-like contact of Ni to the p-type GaAs NWs. (PDF 24 kb)
