# Supplementary material for: Growth and Photovoltaic Properties of High-Quality GaAs Nanowires Prepared by the Two-Source CVD Method
Source: Nanoscale Res Lett. 2016 Apr 12;11:191. doi: 10.1186/s11671-016-1420-y (PMC4829565; doi:10.1186/s11671-016-1420-y)
Supplement: Additional file 1: Figure S1. — I DS-V DS curves of the GaAs NW array FET illustrated in Fig. 3b of the main text. The curves confirm the ohmic-like contact of Ni to the p-type GaAs NWs. (PDF 24 kb) [file 11671_2016_1420_MOESM1_ESM.pdf]

Supporting Information

Growth and photovoltaic properties of high-quality GaAs nanowires prepared by the two-source CVD method

Ying Wang,[1,2] Zaixing Yang,[3,4,5] Xiaofeng Wu,[1,2] Ning Han,[1,2,*] Hanyu Liu,[6] Shuobo Wang,[6] Jun Li,[6] WaiMan Tse,[3] SenPo Yip,[3,4,5] Yunfa Chen,[1,2] and Johnny C. Ho[3,4,5,*]

[1] State Key Laboratory of Multiphase Complex Systems, Institute of Process Engineering, Chinese Academy of Sciences, Beijing 100190, China.
[2] Center for Excellence in Urban Atmospheric Environment, Institute of Urban Environment, Chinese Academy of Sciences, Xiamen 361021, China
[3] Department of Physics and Materials Science, City University of Hong Kong, Kowloon, Hong Kong
[4] State Key Laboratory of Millimeter Waves, City University of Hong Kong, Kowloon, Hong Kong
[5] Shenzhen Research Institute, City University of Hong Kong, Shenzhen 518057, China.
[6] Beijing National Day School, Beijing 100039, China.

* Author to whom correspondence should be addressed.
Electronic mails: nhan@ipe.ac.cn; johnnyho@cityu.edu.hk

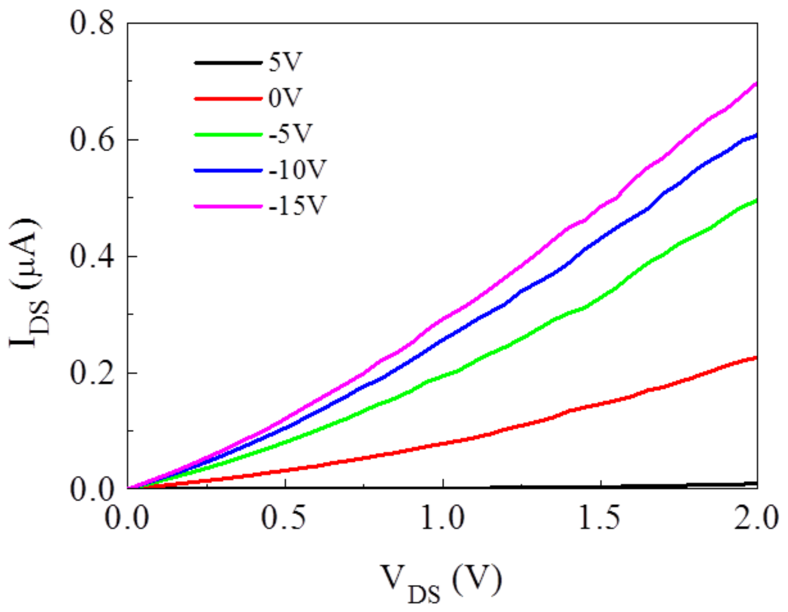

Figure S1. I_{DS}-V_{DS} curves of the GaAs NW array FET illustrated in Figure 3b of the main text. The curves confirm the ohmic-like contact of Ni to the p-type GaAs NWs.
